# Free school meals as an opportunity to target social equality, healthy eating, and school functioning: experiences from students and teachers in Norway

**DOI:** 10.29219/fnr.v65.7702

**Published:** 2021-07-09

**Authors:** Kristine E. Illøkken, Berit Johannessen, Mary E. Barker, Polly Hardy-Johnson, Nina Cecilie Øverby, Frøydis Nordgård Vik

**Affiliations:** 1 Department of Nutrition and Public Health, University of Agder, Kristiansand, Norway; 2 Department of Health and Nursing Sciences, University of Agder, Kristiansand, Norway; 3 Medical Research Council Lifecourse Epidemiology Unit, University of Southampton, Southampton, UK; 4 NIHR Southampton Biomedical Research Centre, University Hospitals Southampton Foundation Trust, Southampton, UK

**Keywords:** free school meal, lunch, Norway, intervention, interviews, social inequality, learning, social environment, diet, school function

## Abstract

**Background:**

There are no national arrangements for free school meals provision in Norway despite this being an important opportunity to improve children’s and adolescents’ nutritional status and ultimately their physical and cognitive development. During a one academic year (2014–2015), a group of Norwegian sixth graders were served a free healthy school meal in a project called ‘The School Meal Project’.

**Objective:**

To explore students’ and teachers’ experiences of receiving free school meals after the free school meal in 2015 and 5 years later.

**Design:**

In-depth, semi-structured interviews with separate groups in 2015 and in 2020 were conducted face to face or via telephone or digital platforms. The findings are based on 13 students (aged 12–16) and 5 teacher interviews.

**Findings:**

Thematic analysis identified four main themes that describe the perceived benefits of receiving free school meals: 1) the meal as a social event where students made new friends and learned new skills; 2) as an aid to forming healthy eating habits; and as an opportunity to 3) improve school functioning and 4) increase social equality among students.

**Discussion:**

Our analysis suggests that the free school meal may influence healthy behaviors not only at the individual level but also at the social-, physical-, and macro-levels. Methodological limitations, including self-selection bias, should be considered when interpreting our findings.

**Conclusion:**

This study provides unique insights into the social benefits for students of receiving free school meals. Our findings illustrate the potential of free school meals: eating healthy foods, sharing a meal together, and interaction between students and teachers at mealtime, to promote health, learning, and equality. In order to maximize these benefits through national implementation of free school meals, more understanding is needed of possible facilitators and barriers related to the provision and uptake of free school meals.

## Popular scientific summary

- We provide novel insights into students’ and teachers’ experience with a 1-year free school meal intervention shortly after the intervention and 5 years later.
- The students and teachers felt that the free school meals were beneficial for a healthy diet, social equality, school function, energy to pay attention, social interaction, and social learning.
- Action should be taken to investigate viewpoints of stakeholders and facilitators and barriers related to the implementation and uptake of free school meals.

Across Europe, school meals are becoming increasingly important target for public health programs. European policymakers widely agree upon school food policy objectives; school food should improve child nutrition, facilitate the development of healthy eating habits, and reduce or prevent obesity (1). Although it is well-documented that provision of healthy school meals improves the nutritional quality of children’s diets in comparison with meals brought from home (2–7), schools across Europe do not yet offer healthy meals to all students (8). Different school meal arrangements exist across Europe. These range from universal free school meals provision as seen in Sweden and Finland (9), free school meals offered to students in low-income households as in UK and Lithuania (10, 11), meals brought from home as is common in Denmark and Norway (9), going home for lunch as is common in Germany and Switzerland (12, 13), or a combination of packed lunches and eating at home as seen in the Netherlands (14). In Norway, there are no national arrangements for provision of a free school meals, and the majority of Norwegian students bring packed bread-based meals from home (15). Challenges with the school lunch in Norway are that many children watch a screen during mealtime, few students bring fruit and vegetables, and some students does not have a packed meal with them (15).

In 2016, the Lancet ‘Commission on Adolescent Health and Wellbeing’ highlighted the importance of investing in young people’s health; it brings a triple dividend of benefits to young people’s health now, as they progress into adulthood, and to their future children (16). Indeed, focusing on facilitating healthy eating patterns in the early stages of life, a time when habits are formed increases the likelihood of sustaining a healthy dietary pattern (17). A healthy diet during adolescence is important for brain health and cognitive development (18). Improving nutritional status during adolescence can, in the long term, lower the risk for cardiovascular and metabolic diseases (17). It has been estimated that globally, one in five deaths could be prevented through improvements in diet (19). Healthy school meals have the potential to impact both these health outcomes as they make an important contribution to students’ total daily energy intake (10, 20).

Evidence suggests that providing free school meals can contribute to an overall healthier diet, especially for students living in socioeconomically disadvantaged households (4, 5, 21). Free school meals have been linked to increased fruit and vegetable intake, and improved attention and energy in students. Universal free school meal provision has also been found to reduce stigma associated with means-tested eligibility for free school meals (22–24). Furthermore, free school meals have the potential to promote a varied diet, as students are given the opportunity to try new foods and dishes. There is some suggestion that they also provide a setting where students have the opportunity to acquire social skills by enjoying a meal together, and thus experience an improved school environment (25).

Between 2014 and 2015, 55 sixth-grade students (11–12 years old) from a Norwegian primary school were provided a free school meal every day during the school year in a project called ‘The School Meal Project’. The project also had a control group (*n* = 109) who continued with eating their packed meals as before (5). Findings from the ‘The School Meal Project’ project showed that students receiving the free school meal had a more varied diet through increased intake of fish, fruits, and vegetables compared to the control group (5, 23). Overall, the intervention in students quality of diet had improved at 1 year follow-up, and this was particularly true of students with lower socioeconomic backgrounds (5).

## Aim

The study reported in this paper was carried out in order to understand how students experienced the free school meal, and the impact it had on their diets and other aspects of their lives both immediately and 5 years later. It also aimed to explore teachers’ experiences of the free school meal. The findings are presented here as they address the following question: How did students and teachers experience the free school meal?

## Materials and methods

### The school meal project

The School Meal Project was an intervention study that investigated the effect of providing free school meals across one academic year. Students (*n* = 55) aged 11 years old at a Norwegian primary school in a rural area in southern Norway were served a free healthy school meal, which met Norwegian dietary guidelines at lunch during the school year 2014–2015. A local cook provided and funded the school meals during the intervention. A few local sponsors also contributed.

The free school meal consisted of whole-grain bread, a variety of healthy foods and fruit and vegetables that were served on large platters in the classroom (5). Yogurt was served on some occasions. Students helped themselves to the food they wanted and prepared the classroom for lunch by organizing their desks so that they sat around one or two tables consuming the meal together. A teacher or an assistant was present during the school meal (5). When there was no intervention (both before and after the study period), students consumed their packed lunch from home alone at their desks in the classroom, usually while watching a screen (such as YouTube videos) or listening to a teacher reading a book. A control group (*n* = 109) continued bringing packed meals from home as usual. The intervention was evaluated using both quantitative and qualitative methods with the findings from the former reported elsewhere (5, 23, 26). This paper reports an analysis of interviews with students and teachers that were carried out immediately post-intervention (2015), and follow-up interviews with teachers and students carried out 5 years later in 2020. In-depth interviews were chosen as the method of data collection in order to encourage participants to reflect upon their thoughts, feelings, and experience of, the free school meal intervention (27). The project received ethical approval from The Norwegian Centre for Research Data in 2015 and 2020 (reference number: 38980 and 514675, respectively). This article was structured using the ‘COnsolidated criteria for REporting Qualitative research’ (COREQ checklist) to ensure transparency in describing methods and findings (28, 29).

### Subjects and recruitment

In 2015, after the end of the free school meal and at the beginning of a new semester, the project leader sent out information and consent letters inviting students and teachers to participate in interviews. Students brought the letter home to a parent/caregiver who signed the consent letter, and then students had to return the consent letter back to the school. The letters were then collected by project workers. Of the 55 eligible students and 5 eligible teachers, 7 students and 3 teachers consented to participate in interviews.

The participants in 2015 and 2020 were two separate groups. The identity of those who were interviewed in 2015 was neither recorded, except for one of the teachers, nor asked (in 2020) if they had participated in interviews in 2015. It was therefore not possible to follow-up the same group at the two time points.

In 2020, students were in their first year of upper secondary school, and therefore not in the same class anymore. Their primary school which participated in ‘The School Meal Project’ had access to their previous registered address from lower secondary school. Project worker KEI delivered the letters to the school, and the school assisted in the recruitment process by sending out letters including project information and consent forms. These letters were sent out to the former students, asking them if they would be willing to be interviewed about their experience with ordinary school meals and with the free school meal they received 5 years earlier. They contacted project worker KEI by telephone for signing up for interviews. Teachers were recruited by e-mail sent from the school head teacher. In total, six students and two teachers who had taken part in the intervention in 2015 agreed to be interviewed in 2020. All participants were 16 years or older at the time of recruitment in 2020. After consulting with the Norwegian Centre for Research Data, it was decided that the students were competent to consent to participating in this research, and permission from parents was not therefore sought. Verbal consent was audio-recorded at the time of the telephone and digital interviews. A written informed consent was obtained from face-to-face meetings.

### Data collection

Interview guides included questions that focused on exploring students’ and teachers’ experiences and their perceptions of the importance of the school meal for their diet, social environment, and learning. The interview guide was pilot tested at both time points on one student in 2015 and one student in 2020, at the same age as the recruited students both years, and a few changes were made considering rewording and structural changes in 2020. Only the relevant items from the interview guide in 2015 related to the aim in this present study are included (Table 1). Items in the interview guide from 2015 evaluating the organization and the implementation of the free school meal (such as student involvement in organizing the meal and practical collaboration) and items measuring student–teacher relations (such as if the student knows what the teacher expects and how teachers communicate) are not presented in the current study.

**Table 1:** Relevant items from the interview guide in 2015.

	Students	Teachers
1	What is your perceptions of the free school meal? *Positive? Negative?* *Any changes in your packed meals now after the free school meal?*	How well did the organization of the school meal work? *Positive? Negative?* *What would you do different?*
2	Challenges during the free school meal? *What challenges did you face and how did you solve them?*	What are your perceptions of the free school meal? *Positive? Negative? What differences did you observe?*
3	Did the school meal lead to any changes in your class? *Positive? Negative?*	How do you perceive that the students experienced the school meal?
4	What is your main experience from the project?	What was your role as a teacher during the free school meal?
5	Belonging and friendship: *Are there any changes in who you spend time with? And if so, what changes?* *Do you believe that the school meal can impact who you spend time with? If so, how?* *Do you talk more/less when you share a meal together?* *How can a shared meal impact how you behave with one another?*	How can a free school meal impact the social climate in the class? *Did you observe any changes in who the students preferred to spend time with?* *Did they talk more/less during a shared meal?* *Differences in how the students behaved with one another?*
6	What did you learn from the project? *What value do you believe school meals have?*	How can a shared meal impact student learning? *Did you observe any new sides of the students?* *Changes in their concentration, motivation, activity in class, or general behavior during the free school meal?*
7	Comments/other things you would like to add?	Challenges during the free school meal? *What challenges did you face and how did you solve them?*
8		Did the school meal lead to any changes? If so, what changes? *Positive? Negative?*
9		What did you learn from the project? *What value do you believe school meals have?*
10		Comments/other things you would like to add?

Questions in ***italic*** illustrate example of prompt questions.

In 2020, the interview guide was developed by KEI, a PhD student. Members of the research team (FNV, BJ, and NCØ) reviewed the guide and provided suggestions for improvement (Table 2). The interviewers were KEI in 2020, and two master students in 2015, all women (aged 20–30 years) with a public health background. Participants in the study did not have a prior relationship with the interviewers. The interviews were audio recorded and carried out with only the interviewer and participant present.

**Table 2:** Interview guide 2020.

	Students	Teachers
1	Tell me about your experience with school meals *Experiences from the free school meal 5 years earlier?* *Experience from school meals besides the free school meal?*	Tell me about your experiences with the free school meal *What do you remember?*
2	What do you eat for school meals now and how has this changed during your years at school?	How did you experience the importance of the free school meal for *The social environment, diet, behavior, concentration, learning, you teachers?*
3	What did you eat for the free meal in 2015? *What was different with the free school meal compared to your packed meals?*	What would you do differently? *Which challenges can you identify with a free school meal?*
4	What do you think about free school meals? *Positive? Negative?*	What do you think about free school meals? *Positive? Negative?* *What worked well in 2015?*
5	Which importance do you believe the free school meal means for: *The social environment, diet, behavior, concentration?*	If you decided, how would the school meal look like? *Organization and content of the meal?*
6	If you decided, how would the school meal look like? *Organization and content of the meal?*	Comments/other things you would like to add?
7	What would you do differently in the school meal project?	
8	What worked well in the school meal project?	
9	Comments/other things you would like to add?	

Questions in ***italic*** illustrate prompt questions.

All interviews in 2015 were conducted face to face at the school, and their duration was between 25 and 46 min. Interviews in 2020 with students were conducted over the phone, face to face, or as digital interviews and were between 20 and 40 min long. The teacher interviews in 2020 were conducted digitally using the platform Zoom because the COVID-19 pandemic made face-to-face meetings impossible. Participant characteristics and interview methods are given in Table 3. In 2020, students and teachers were sent a gift-card on 250 NOK (≈25 EUR) following the interviews.

**Table 3:** Participants characteristics (*n* = 18).

	Student 2015 (*n* = 7)	Student 2020 (*n* = 6)	Teacher 2015 (*n* = 3)	Teacher 2020 (*n* = 2)
Boy/man	4	4	1	1
Girl/woman	3	2	2	1
Telephone	0	4	0	0
Digital video	0	0	0	2
Face to face	7	2	3	0

### Analysis

The interviews were transcribed verbatim, and transcripts were uploaded to NVIVO for data management. A thematic analysis following Braun and Clarke’s guidelines (30) was carried out using a combined deductive and inductive approach. The deductive approach was used to produce a first analysis of the data based on the research questions and the topics in the interview guide; inductive analysis permitted identification of themes underlying what was said in the interviews and allowed new ideas and interpretations to emerge from the data. The transcripts from interviews in both 2015 and 2020 were read, reread, coded, and recoded by two research team members: KEI and FNV. Codes were compared, and discrepancies were discussed and resolved. Table 4 shows a section of the coding frame, with themes, codes, and illustrative quotation.

**Table 4:** Examples of quote, code, and main theme from the coding frame.

	Code	Main theme
The students were less concerned whether they had brought a packed meal when they were served a free school meal (teacher interview)	Availability of healthy food	School meals as an opportunity to improve equality
My brain function and alertness improve if I eat healthy	Improved function	Improved school functioning

First, the data from 2015 to 2020 were analyzed separately. We then compared the data looking for differences and similarities across the groups and concluded that the results should be presented together as the themes from both sets of interviews were similar. The few exceptions to this are also presented. The themes were not considered as definitive until the data had been read and coded at least twice by two members of the research teams, and when there was agreement that the data were organized in a meaningful and useful way (29). The findings of this analysis are presented as they answer to each theme, using direct quotations with participant pseudonym to illustrate.

## Findings

The overarching themes described the benefits of receiving free school meals from the perspectives of both students and teachers. Participants viewed the free school meal as a social event where they could make new friends and learn new skills and considered the free school meal to have a positive impact on quality of student diet, school functioning, and social equality (Table 5).

**Table 5:** Experiences of receiving free school meals according to year and participant group.

Main theme (in bold) and subthemes	2015		2020	
	Teachers	Students	Teachers	Students
School meals as a social event	X	X	X	X
Increased social learning	X		X	
School meals as potential for forming healthy habits	X	X	X	X
Improved school functioning		X		X
School meals as an opportunity to improve equality			X	X

### School meals as a social event: making new friends and learning new skills

Many of the participants at each stage of interview discussed how the free school meal was beneficial in terms of social functioning at school. As the students, during the intervention, moved from eating packed meals alone at their desk in the classroom watching video or listening to a teacher reading a book, to sitting together and sharing the meal, they experienced increased interaction. This increased interaction was mentioned both in 2015 and in 2020. Teachers stated that eating the free school meals together created a comfortable atmosphere during which students became more friendly toward each other. By sharing a meal together, they got to know each other better and experienced increased social inclusion. In 2015, some students even said this was important for making new friends, illustrated by Christopher:

‘… when we talk to each other, we get to know more about one another. We know each other better and then I start to become friends with them’.

Although students’ social experiences during lunchtimes were mostly described as positive, some negative experiences were reported. For example, the teachers described more noise during the lunch break as the students sat together and talked loud to each other. Furthermore, in 2020, some students remembered that their peers commented negatively on what they were eating. As Penny, a student talked about:

‘You were afraid of eating fish spread even if you liked it, because others would say that it was unpleasant. That it was smelly…’

Teachers would typically correct unwanted behavior, as Hannah, a teacher said in 2015:

‘The thing is, they actually had to learn to not make a big deal out of it if someone opens a fish spread, right. Don’t make a big deal out of it because it is actually bullying’.

Some of the students felt inhibited by such comments and stopped eating such foods.

School meals as an opportunity to learn social skills

This theme derived mainly from the teachers. Teachers experienced the students in a new setting and got to know them better. The teacher Hannah illustrated this in a conversation in 2015:

‘Some of them actually started talking more. They used to be quiet [during class] … And, some students that seemed polite and kind, or just dutiful, some of them were almost a little rude. Right, so you could see, there were some things they could not actually do. Like sharing and passing [food] and stuff like that. They are good at school, but they were actually a little silly at the table … These are not things you see when you have them in class. You could see it during the meal’.

From the teacher’s perspective, free school meals were an important opportunity for the students to learn how to talk and interact with one another, as illustrated above in the example with the fish spread. Students practiced table manners, learned about food culture, and learning, for example, to show gratitude and to be polite when they were served food. Examples of this were given by Kate in 2015 when she was asked about the teacher role during the meal:

‘… That you [teach them to] talk in a calm way, that you don’t shout at the table, that you can’t only think of yourself when you’re taking food … That they learn to stand in queue’.

In 2020, when asked to elaborate on her thoughts on the free meal and learning, the other teacher Hannah commented:

‘It’s what I said about ordinary table manners. Sharing, to see each other, right? … The meal is not as important in the Norwegian culture as it is in other cultures, right? But there is a lot of learning from sitting together and talking together. To see each other … and to see that there should be enough [food] for everyone. To not be greedy. To say ‘thanks’ or ‘can you pass me ...?’ These are things we take for granted, but that they have not actually practiced. They have to learn to see that ‘wow, it is really nice that there is enough for everybody and not just me’.

The importance of the free school meals as a potential platform for learning social skills should in the view of the teacher Hannah outweigh the extra workload they generate for teachers. Indeed, she suggested that involving students in organizing the meal might reduce the workload for teachers.

‘… In a busy school day, there are many teachers that are thinking ‘is there yet another thing we have to think about now?’…But what you are not thinking of, is the little things they actually learn from a meal like this. That … it is like wearing a uniform, everybody eats the same food, that it is actually important, that they are equal …. In my opinion, if this [free school meal] continued, it should be a school thing, with high student involvement, organized in a way that brings learning’.

Only one student, James in 2020, talked about how the free school meal made them improve their behavior at the table:

‘You learn table manners …, you really have to behave, … especially when you know somebody made an effort to prepare the meal for us’.

### School meals as a potential for forming healthy eating habits

At both sets of interviews, students expressed that the free school meal encouraged them to eat more healthily. Specifically, they suggested that the exposure to fruit and vegetables in the free school meals increased their liking of them. An example of this was seen in a student interview with Leon from 2020:

‘I started eating more bell pepper and cucumber. I did not like it before I started eating it at school. But then, I ate it at school almost every day for about one year, and then I started liking it’.

Interviewer: ‘Do you still eat it?’

‘Yes I do. Every day’.

Several students even traded out their white bread and chocolate spread for whole grain bread, cheese, and salad, illustrated by Christopher in 2015:

‘Before the food project, I always had “Nugatti” [chocolate spread] and stuff -but after … the food project and for a while afterwards, I started to eat more healthy food’.

It was important to the students that they had some choice about what they ate, and that they had the chance to eat varied foods and to eat until they felt full. Carl, in 2020, said that:

‘Some students might bring a packed meal, or it might be prepared by a parent, and they do not want to eat it. The parents might think that they eat it, but it can get thrown away in the garbage. When you received it for free, you can decide yourself what you want to eat … For me, I ate more. I believe this was a good thing for me, because I was thin when I was younger’.

Furthermore, they preferred food that was fresh and visually appealing, in contrast to the packed meal that was in their backpack for 3–4 h before they ate it. Sarah, in 2015, talked about that:

‘It was a difference in the amount of food I ate when we received it for free. Before, I ate one slice of bread, maybe two. When we received it for free, I could eat 3–4 slices of bread. We could eat nice, fresh cheese, instead of the sweaty cheese in my packed meal. Or, like with banana, it would get brown’.

Many of the students in 2020 also remembered that the food was fresh and that this was a part of why they liked it. Carl, in 2020, said that:

‘It was different food, and we knew that it was fresh.. what made the packed meal unappealing, was that we usually eat the same every day. Or that it could take 3–4 hours from we prepared it until we ate it’.

Students interviewed in 2015 believed that the free school meal increased their knowledge of healthy eating. Students interviewed in 2020 did not mention this in their interviews. Rachel, in 2015, stated that:

‘It is just as tasty with healthy [food] as it is with unhealthy, and it is better to eat healthy. So, I think that many started to realize, or some of them they understood that healthy [food] was much better’.

Teachers also perceived a change in students’ eating habits as a consequence of the new social dynamic at mealtimes. Seeing what their peers were eating encouraged some students to try new food. This is illustrated by teachers Hannah in 2020:

‘In the beginning, some of them had their own packed meal with white bread and chocolate spread. After a while, this became strange. It did not fit within the group…’

and Kate in 2015:

‘I remember when we started with the school meal project, that some of them did not like whole wheat bread …But when the school meal project started, they had to eat the bread. So, they ate it, something they learned during the school meal project’.

### School meals as an opportunity for improved school functioning

Both in 2015 and 2020, students reported that free school meals increased their concentration and enabled them to have energy throughout the rest of the school day. Sarah, a student in 2015, said that:

‘I believe we were more active in class the last semester’.

Interviewer: ‘Why do you believe you were more active in class?’

‘Maybe it was because we had eaten and that we were more used to eat healthier …’

Later in the interview, the interviewer asked:

‘Did you learn anything from being a part of the school meal project?’

Sarah answered: ‘I learned that you can improve your concentration by eating healthy…’

The students stated that they felt more awake and paid more attention, and the teachers reflected that students took a more active role in class. Ron, a student in 2020, said that:

‘The food was good, it was fresh and tasty. I don’t think anybody disliked it.… It [the free school meal] … made you more awake, and ... actually gave you energy’.

Interviewer: ‘Why do you believe it is important to have food that gives energy and makes you awake?’

‘Because when you sit there in class …it is easy to lose focus and doze off. But if you have a really good meal and eat until you are full, then you feel more awake, you improve your concentration, and can actually start to pay attention. At least I noticed that… Before 6th grade, I ate a lot of chocolate spread. Yes, I was, like, less able to concentrate. Like, felt tired’.

Free school meals were also considered to be important for a calmer learning environment during class, especially after the meal break. Instead of focusing on how hungry or unwell they felt, they could focus on the class activities, and the students spoke about the fact that they were not as hungry as they used to be after the school ended. Paula, a teacher in 2015, describes how important this is during class:

‘If they don’t eat at school, the [school] performance will decline. If they are hungry … they can’t stop thinking about it. It gets all the attention’.

### School meals as an opportunity of reducing inequalities between students

The potential for free school meals to reduce social and health inequalities between students was an important theme in the conversations with students in 2020; they talked about how important the free meal was for children who did not eat lunch or whose parents could not afford to give them lunch. Students were worried about the availability and affordability of healthy food in such households. As Leon said about the free school meal:

‘What worked best was that everybody got to eat food. Not everybody ate before we received the free school meal (…) It [free school meals] was positive for, like, not everybody can afford food. So, it would be nice to be served free food at the school’.

Furthermore, the students talked about how important it was to eat the same food, to feel equal and not compare the contents of packed lunches from home, illustrated by Carl:

‘In my opinion, it was a very good thing that everybody got the same food …. [Before] we could see that … some people brought, for instance, a chicken salad. You could see that some people were better off than others. At least, the food looked better’.

In an interview with the teacher Hannah from 2020, she commented that the free school meal equalized the students’ food experiences and that because of this, the poorer students could relax during the meal.

‘I am aware of what they had in their packed meals, but they did not always show it to the others. I think, that with the [free] meal, they could relax and eat what everybody else was eating. I believe this was important to their wellbeing. The fact that nobody can see that I had the worst bread, maybe it was moldy… Some are almost embarrassed because they had a store-bought sandwich or a pack of pastries… Like, for some it was embarrassing that they did not have a nice, packed meal’.

Discussions of the impact of free school meals in reducing social inequalities were more a feature of the interviews carried out in 2020, suggesting that this benefit became more obvious and important to students as they matured and reflected on the experience. Leon said that:

‘I don’t know if it was necessarily hugely relevant in my class, but when I think of Norway at large, it is not a given that everybody can afford a good, packed meal every single day’.

## Discussion

The aim of this study was to explore how students and teachers experienced the free school meal and what they perceived as its benefits and challenges both immediately after and 5 years later. This study found that free school meals offered an arena for making new friends and learn social skills, as an aid for forming healthy eating habits, and an opportunity for improving school function and increasing social equality, thus viewing the free school meal as a positive impact on their school life and health. Furthermore, our study suggests that free school meals may represent an opportunity to support students in their social development as well as to improve their diets and capacity for learning. Indeed, school meals have gained more attention the past decade for their potential as a pedagogical tool contributing to learning about nutrition, sustainability, culture, and social- and political systems (25, 31, 32). Oostjinder and colleagues (31) give an example of how school meals can also influence public health and sustainability through interaction with education, food, environment, social relationships, policies, and to produce optimal and sustainable food behavior.

The findings from this study suggest that the learning of social skills during free school meals may be maximized if teachers play a role in managing the eating experience. Previous research has also shown that the interaction between students and the teacher responsible for supervising the meal is important for creating learning during the meal (25). Research indicates, however, that there is a tension between school meals as a pedagogic situation and the school meal as a break for students; an opportunity for them to occupy their own space without an adult agenda (33). The study reported here highlights the possibility for free school meals to be both, with students enjoying the meal together while also learning how to behave and talk to each other from interactions with the teacher and other students.

As in previous research, this study found that free school meals can create a feeling of equality and solidarity (34, 36). It seems important that the meal is served as a universal free school meal, as free meals offered to students with low socioeconomic status (SES) can generate stigma and a sense of segregation (22, 36). This was seen in our study illustrated in comments from students who were well aware that some other students were unable to afford good quality packed lunches, and in the experiences of a teacher who noticed the embarrassment of children with poor quality packed lunches. This study adds new knowledge in that students remembered 5 years later how it felt to be equal, something that was much clearer for them in retrospect. This highlights how students seemed to value social equality as an important aspect of free school meals.

Free school meals have, as mentioned previously, also been found to improve the quality of young people’s diets especially those of low SES young people (5, 21). An explanation for this may be that low-income families might be more reluctant to purchase unfamiliar foods and risk waste. Parents with low income tend to buy foods they know children like, often calorie-dense, nutrient-poor food, to reduce food waste (37). This denies children the chance to try unfamiliar foods and may prevent them from forming new tastes since repeated exposure is known to be important in forming new taste preferences (38). Our findings are in line with those from Benn and Carlsson (25), which show that the free school meal offered, to everyone regardless of income, a variety of foods and facilitated repeated exposure to new tastes. In addition, it is likely that students’ were influenced by eating with classmates and may have tried new foods because others were eating them (39). This may be partly responsible for the perception that social equality was enhanced, and diets improved by the free school meal in our current study.

Students in this study claimed they felt better able to concentrate, had more energy, and improved learning when they were served a free school meal. This is not surprising, as it is line with previous research, indicating a link between free school meals and cognitive performance, connecting food and meals to improved concentration, reading performance, attention, fullness, and readiness to learn (22, 25, 40).

During lunchtimes throughout the free school meal, both students and teachers perceived that interaction levels between the students increased. For the most part, this contributed with social benefits such as getting to know each other better and making new friends. This is supported in previous research showing that free school meals can create a good classroom atmosphere by sharing and enjoying a meal together (25). At the same time, the increased interaction also led to increased noise during the lunch break and negative comments. It appeared evident with teacher involvement in the lunch break to stop and correct this negative behavior. In line with previous research (25, 41), we recommend teacher involvement in future free school meal programs as their role appears to be important in creating a good atmosphere and for social learning, thus increasing the learning potential of school meals.

To our knowledge, negative findings relating to free school meals have mainly been related to poor quality of meals and lack of variation and autonomy (25, 34, 42, 43), stigma or segregation for student with low SES (22, 36, 44, 45), and organizational factors as, for instance, standing in line and having too short time for eating (31, 43, 46). None of this was described by the students and teachers interviewed in the study reported in this paper.

Even though there were many similarities in how teachers and students experienced the free meal program, some differences were identified. As mentioned above, students in 2020 were 5 years older, and therefore more prone to reflect on free school meals in relation to social equality. To our surprise, most of the students, in contrast to teachers, stated that the free school meal contributed to their improved function at school. For teachers, it seemed more important to talk about how the free school meal was beneficial for promoting social skills among students. Increased social skills and a socially inclusive environment are important, as Hale and Viner (47) identified an association between social exclusion and poor education and employment outcomes. This suggests that interventions such as free school meals offered as they were in our project might result in improved educational-, employment-, and health outcomes later in life. Findings from this present study are consistent with previous research, showing great potential of free school meals (4, 5, 22).

### Free school meals as a socioecological intervention

Our findings indicate that a free school meal may achieve improvements in diet, health, and well-being through action at multiple levels. Our diets and health are the product of an interplay of individual factors (cognition, skills, biological, and demographic factors), the social environment (role modeling and social support), the physical environment (availability of healthy food), and macro-level environment (social and cultural norms, and policy) (48). Free school meals can be viewed as an intervention that exerts its influence at all these levels and requires action to be taken at all these levels. Students in our study claimed that the school meal increased their liking and intake of healthy food, indicating a change at the level of the individual which improved their diets. In addition, the school meal improved their school function, with increased concentration, energy, and social skills. Improvements in their diets and school function were also influenced through changes to the social environment, whereby increased interaction was necessitated by sharing food with other students at lunch times, changes to the physical environment through the provision of healthy food and creation of meal-time environments which supported interaction and learning. As an intervention to support learning healthy habits, the free school meal may be seen as one way of changing social and cultural norms governing healthy eating, and of delivering European school food policies which aim to improve nutrition and teach healthy habits to young people (1). As a small qualitative study, our research can only indicate these potential benefits from free school meals. Larger trials of the long-term impacts of free school meal provision at all these levels are needed to establish whether they are effective in achieving public health improvements.

### Strengths and limitations

There were potentially 55 participants able for recruitment in this study, but it was not possible to contact them directly as their contact information from 2015 was deleted after the intervention. As for needing the school to assist in the recruitment, and the participants chose whether to participate, we do not know how many that received the invitation in 2020. The voluntary participation, which is a fundamental research ethics principal, constitutes a risk of self-selection bias, a risk for the study sample to differ from those who did not participate. Participants who sign up for interviews can, therefore, be more open and more interested in the research topic (49). Furthermore, there is a probability for the students who participated in interviews in 2020 to not be especially vulnerable, as more psychologically vulnerable young people might hesitate to take initiative and contact a stranger to volunteer in interview-based research (50, 51). Thus, it could be that participants who signed up for interviews particularly remembered and liked the free meal, that they were motivated by receiving the gift card, or that they were reluctant to sign up, and therefore not accurately reflecting the view of all the students who received the free school meal. As 5 years is a long time, especially for children, there might be a risk for recall bias that could impact their memory as well. Furthermore, some of the informants, particularly the teacher Hannah, were more elaborative in their responses to questions, resulting in longer, more detailed interviews, and therefore more quotes than the other teachers. Based on Hannah’s interviews, it seems clear that this teacher was positive toward free school meals. This might give undue weight to her opinions and experiences.

Differences in data collection methods are likely to alter the duration and depth of the interviews. Telephone interviews tend to be shorter compared to face-to-face methods, and participants tend to provide less detail over the telephone (52). This might be the case for our study as there was a combination of different data collection methods. Moreover, students may have been influenced by the interview guide, and the items from the 2015 interview guide that we did not present in the current study might have affected the tone and duration of the interviews. There were also distractions during three of the interviews, one having noise at school and two having bad reception over telephone interviews, which could lead to misunderstandings. However, the interviewer asked back to get their answers confirmed, and statements that were unclear when transcribed were excluded from the analysis.

This was a small study that might raise questions about the possibility of data saturation. Braun and Clarke (53) have questioned the assumption that large numbers of participants producing more valuable data and claiming data saturation are necessary for validity. Indeed, we identified few dissenting viewpoints in the student interviews, which we expected: students had overall positive experiences related to the free school meal. Furthermore, they did not have to stand in a long line for food, they had a variety of food choices, and it was free for all, all of which previous research has identified as negative with free school meals. We reached the point where after several interview, no new themes were emerging, and therefore we feel that, based on student interviews, data saturation was likely to be met. However, we did only interview four teachers, and the teacher ‘Hannah’ indicated in her quote that many teachers are busy (e.g. ‘many teachers that are thinking “is there yet another thing we have to think about now?”’). There is therefore more likely that dissenting views among teachers and other stakeholders such as school leaders and food providers exist. Furthermore, these views are likely to affect the implementation of free school meals and should be investigated in future research.

In relation to the study described in this paper, we argue that the value of the interviews is in understanding in a detailed way the experiences of this small group of informants who were interviewed in two groups 5 years apart, giving us insight into the long-term effects of a free school meal program on young people. In addition, the youth perspective on free school meals is poorly represented studies of the topic. We did not include debriefing or participants checking, as the topic was non-sensitive, and therefore not deemed necessary to do so. However, this may limit the credibility of the interpretation. On the other hand, credibility was enhanced by having two research members systematically reading and analyzing the data (29).

Other strengths of this study include the follow-up interviews 5 years later, something that is rarely represented in qualitative research. The challenges with this study and different school meal traditions and arrangements in different parts of the world may ultimately mean that our study findings may not be transferable to all schools.

## Conclusion

The free school meal was perceived by students and teachers as beneficial for an overall healthy diet and social and health equality. Regardless of income, the free school meal provided healthy, filling meals that students wanted and needed for growth and optimal function at school. A socioecological perspective indicates the multiple levels at which a free school meal intervention operates to promote young people’s health and well-being.

Regardless of free school meals being offered or not, we recommend interaction between teacher and students at mealtimes to enhance student’s social skills. Further research should investigate facilitators and barriers related to provision and uptake of free school meals.
